# ^13^C tracer analysis suggests extensive recycling of endogenous CO_2_ in vivo

**DOI:** 10.1186/s40170-022-00287-8

**Published:** 2022-07-07

**Authors:** Likun Duan, Daniel E. Cooper, Grace Scheidemantle, Jason W. Locasale, David G. Kirsch, Xiaojing Liu

**Affiliations:** 1 Department of Molecular and Structural Biochemistry, NC State University, Raleigh, NC 27695 USA; 2 Department of Radiation Oncology, Duke University School of Medicine, Durham, NC 27708 USA; 3 Department of Pharmacology and Cancer Biology, Duke University, Durham, NC 27708 USA

**Keywords:** ^13^C tracing, High-resolution mass spectrometry, Anaplerotic metabolism, CO_2_ recycling

## Abstract

**Background:**

^13^C tracer analysis is increasingly used to monitor cellular metabolism in vivo and in intact cells, but data interpretation is still the key element to unveil the complexity of metabolic activities. The distinct ^13^C labeling patterns (e.g., M + 1 species in vivo but not in vitro) of metabolites from [U-^13^C]-glucose or [U-^13^C]-glutamine tracing in vivo and in vitro have been previously reported by multiple groups. However, the reason for the difference in the M + 1 species between in vivo and in vitro experiments remains poorly understood.

**Methods:**

We have performed [U-^13^C]-glucose and [U-^13^C]-glutamine tracing in sarcoma-bearing mice (in vivo) and in cancer cell lines (in vitro). ^13^C enrichment of metabolites in cultured cells and tissues was determined by LC coupled with high-resolution mass spectrometry (LC-HRMS). All *p*-values are obtained from the Student’s *t*-test two-tailed using GraphPad Prism 8 unless otherwise noted.

**Results:**

We observed distinct enrichment patterns of tricarboxylic acid cycle intermediates in vivo and in vitro. As expected, citrate M + 2 or M + 4 was the dominant mass isotopologue in vitro. However, citrate M + 1 was unexpectedly the dominant isotopologue in mice receiving [U-^13^C]-glucose or [U-^13^C]-glutamine infusion, but not in cultured cells. Our results are consistent with a model where the difference in M + 1 species is due to the different sources of CO_2_ in vivo and in vitro, which was largely overlooked in the past. In addition, a time course study shows the generation of high abundance citrate M + 1 in plasma of mice as early as few minutes after [U-^13^C]-glucose infusion.

**Conclusions:**

Altogether, our results show that recycling of endogenous CO_2_ is substantial in vivo. The production and recycling of ^13^CO_2_ from the decarboxylation of [U-^13^C]-glucose or [U-^13^C]-glutamine is negligible in vitro partially due to dilution by the exogenous HCO_3_^−^/CO_2_ source, but in vivo incorporation of endogenous ^13^CO_2_ into M + 1 metabolites is substantial and should be considered. These findings provide a new paradigm to understand carbon atom transformations in vivo and should be taken into account when developing mathematical models to better reflect carbon flux.

**Supplementary Information:**

The online version contains supplementary material available at 10.1186/s40170-022-00287-8.

## Introduction

Cellular metabolites are in dynamic homeostasis. Metabolomics is an emerging tool to measure metabolic changes that represent the dynamic status of the cell, tissue, or whole organism and facilitate a better understanding of biological processes from a global level [1]. However, metabolomics mainly measures metabolite concentrations or the changes of metabolite levels, which reflect the net outcome of various metabolic pathways in which each metabolite is involved in, and it does not provide information on individual pathways that contribute to the production and disappearance of a specific metabolite. In contrast, stable isotope tracing provides information on metabolite dynamics and generates data on specific metabolic pathways. Stable isotope tracing has been used to probe specific metabolic pathways in various biological systems since the last century, soon after the initial isolation of isotopic tracers [2]. Over the past decades, advances in instrumentation and data analysis software enabled fast data acquisition and determination of isotope labeling patterns [1]. However, the data from isotope tracing experiments do not provide easily interpretable results, and interpreting ^13^C metabolite labeling patterns remains the limiting step of using stable isotope tracing to address biological questions [3]. To extract maximal information from ^13^C tracing experiments and draw accurate conclusions, it is important to understand which labeling patterns represent different metabolic activities. Understanding the ^13^C transformations can help identify unexpected metabolic pathways of biological significance. For example, [U-^13^C]-glutamine would produce citrate M + 4 following standard glutamine metabolism pathways, which involve glutaminase-mediated conversion to glutamate and then α-ketoglutarate, followed by oxidative metabolism after entering the TCA cycle [4]. The observation of citrate M + 5 suggests the existence of an alternative glutamine metabolism route, the reductive carboxylation of α-ketoglutarate, and the reverse flux relative to the canonical oxidative TCA flux [5, 6]. This pathway has been demonstrated to be important for tumor growth under hypoxic conditions or tumors with mitochondrial defects and may serve as a potential therapeutic target [5, 6]. One challenge of interpreting ^13^C labeling patterns comes from the effects of culturing conditions or tissue microenvironment on cell or tissue metabolism. For instance, factors such as nutrient availability, oxygen level, or tissue context-dependent microenvironment can greatly impact cellular metabolism [7] and consequently isotopic labeling of metabolites. Cells or isolated tissues fed with ^13^C tracers ex vivo also tend to have different labeling patterns from the same type of tissues of animals receiving in vivo tracing [1, 8]. These factors result in difficulties in data interpretation and developing new approaches to decipher labeling patterns.

Pyruvate carboxylase, a mitochondrial enzyme that catalyzes the production of oxaloacetate by combining pyruvate and bicarbonate, is one of the major anaplerotic enzymes. Pyruvate carboxylase supports gluconeogenesis in the liver and kidney by providing oxaloacetate. Pyruvate carboxylase is important for de novo synthesis of fatty acid in adipocyte tissues and is also involved in de novo synthesis of a neurotransmitter, glutamate, in astrocytes. Studies have shown that pyruvate carboxylation is required for tumor growth when glutamine metabolism is suppressed [9, 10]. ^13^C tracing has been used to study TCA anaplerosis in vitro and in vivo, and citrate M + 3 has been used to monitor pyruvate carboxylase activity after [U-^13^C]-glucose tracing [10, 11]. For instance, under cell culture conditions, pyruvate M + 3 can enter the TCA cycle via pyruvate carboxylase and lead to the appearance of citrate M + 3. Indeed, citrate M + 3 was observed in cultured cells fed with [U-^13^C]-glucose. Nevertheless, citrate M + 1 has been reported to be more abundant than M + 3 in lung cancer, liver, and kidney after continuous infusion of [U-^13^C]-glucose or dietary delivery of [U-^13^C]-glucose in vivo [8, 11], but it remains poorly understood why M + 1 is more abundant in vivo. In addition, it was reported that in pancreatic cancer cells [12], both pyruvate carboxylase and malic enzyme 1 activity (pyruvate to malate) contributed to pyruvate carboxylation, further increasing the complexity of labeling patterns.

Here, we report the distinct ^13^C labeling patterns of TCA intermediates in cultured cells, in vivo tumors, and non-tumor tissues after [U-^13^C]-glucose or [U-^13^C]-glutamine tracing. We identified M + 1-labeling of TCA intermediates is the most abundant species in vivo, but not in vitro. This finding is consistent with the results of in vivo [U-^13^C]-glucose tracing performed by other investigators [8, 11, 13, 14]. We hypothesized that endogenous CO_2_ is labeled by ^13^C tracers and subsequently used to produce M + 1 isotopologues of TCA metabolites. To test this hypothesis, we traced the product of other CO_2_-consuming reactions such as purine biosynthesis and measured adenosine M + 1 in tumor and non-tumor tissues. We then compared the time course of M + 1 and M + 3 TCA intermediates and observed that M + 1 citrate appeared earlier than M + 3, which further suggests that CO_2_ is produced endogenously and fixed into the TCA cycle. These findings provide a new paradigm to understand carbon atom transformations in vivo and should be taken into account when developing mathematic models to better reflect carbon flux.

## Results

### M + 1-labeling of TCA metabolites is dominant in the in vivo tumor but not in in vitro cultured cells fed with ^13^C glutamine tracer

**Fig. 1:**
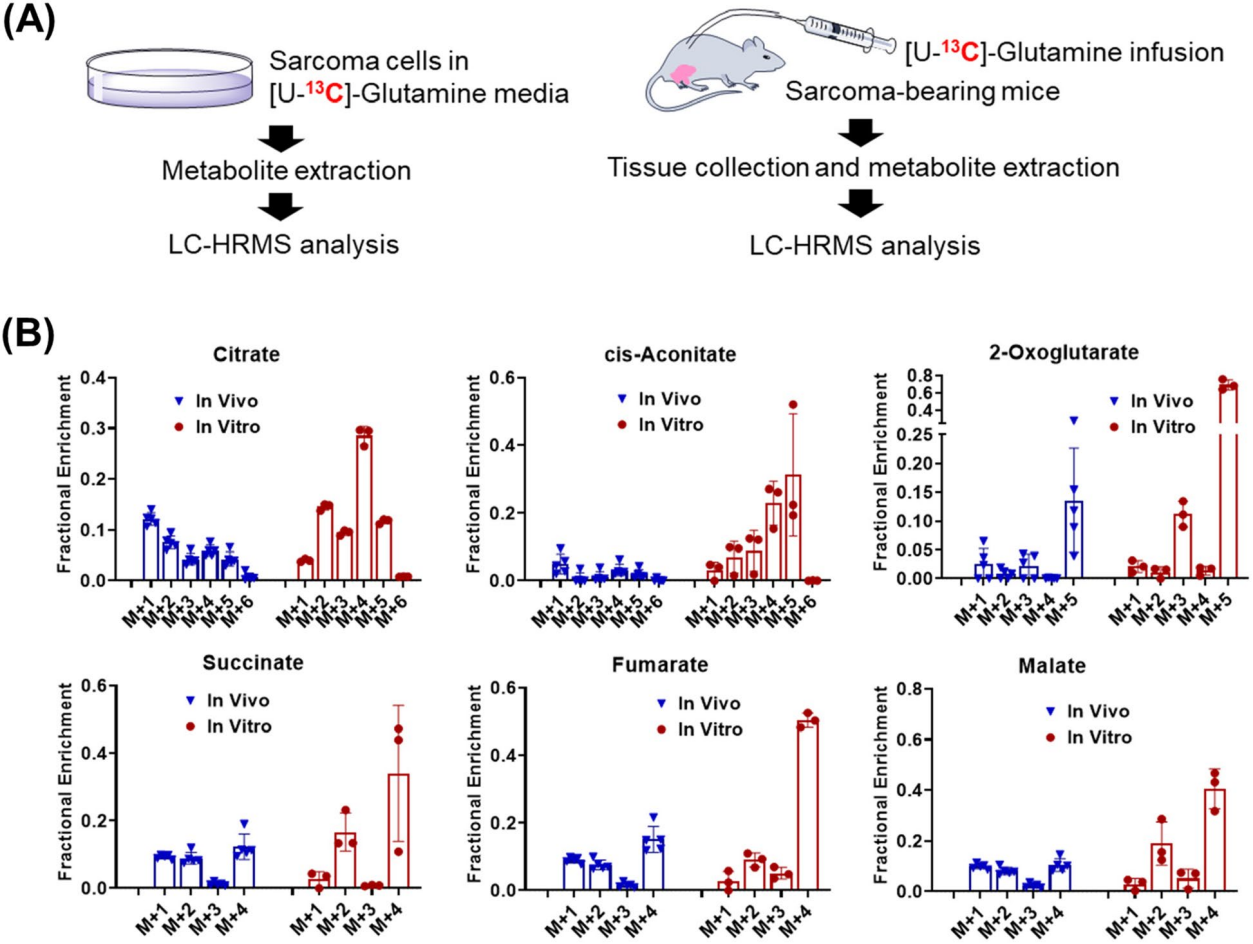
Distinct ^13^C labeling of TCA intermediates with [U-^13^C]-glutamine is observed in in vitro cell cultures and in tumors in vivo. **A** Schematic of in vivo and in vitro tracing analysis. **B** ^13^C labeling patterns of metabolites from sarcoma cells (in vitro) fed with [U-^13^C]-glutamine medium for 3 h (h) and sarcoma tissue from sarcoma-bearing mice (in vivo) receiving [U-^13^C]-glutamine infusion for 3 h. Data are presented as mean ± SD *n* = 5 for animals and *n* = 3 for cultured cells. Data in this figure was after natural abundance correction

To investigate the ^13^C labeling patterns of TCA intermediates in cancer cells (in vitro), and in animals (in vivo) with [U-^13^C]-glutamine, two different types of tracing experiments were performed (Fig. 1A). Sarcoma cells, generated from primary mouse sarcomas, were incubated in RPMI 1640 containing 2 mM [U-^13^C]-glutamine and 10% dialyzed FBS. Intracellular metabolites were extracted after 3 h of tracing and subsequently analyzed using LC coupled with a high-resolution mass spectrometry (LC-HRMS). Sarcoma-bearing mice were infused with [U-^13^C]-glutamine for 3 h. At the end of infusions, sarcoma samples were collected and analyzed using LC-HRMS. ^13^C enrichment was calculated based on metabolite peak area. Natural abundance correction was performed using software R with Bioconductor R package IsoCorrectoR (21). In tumor (in vivo), [U-^13^C]-glutamine was converted into [^13^C_5_]-2-oxoglutarate (M + 5) via glutaminolysis (Fig. 1B), followed by oxidative decarboxylation of 2-oxoglutarate M + 5, resulting in succinyl-CoA and succinate M + 4. The M + 4 carbon chain remains labeled in the next two reactions to produce malate M + 4. Malate M + 4 will be oxidized to oxaloacetate M + 4, which is then used to generate citrate M + 4. The citrate M + 4 was then dehydrated by aconitase to give M + 4 cis-aconitate. Isocitrate dehydrogenase (IDH) catalyzes the reversible reaction of isocitrate to alpha-ketoglutarate and CO_2_, and the reverse reaction contributes to the generation of citrate M + 5 and cis-aconitate M + 5 (Fig. 1B). In the cultured sarcoma cells, M + 4 and M + 5 isotopologues were the major species as expected. Surprisingly, unlike the labeling patterns in cultured cells, M + 1-labeled intermediates were the major isotopologues in mouse sarcomas in vivo. To rule out potential systemic errors of orbitrap-based instruments in detecting lighter and heavier isotopologues, we randomly selected few metabolites detected in sarcoma samples analyzed by Exploris 480 (orbitrap-based mass spectrometers) in full scan mode and plotted the experimental and theoretical values of M + 1 peak (containing one ^13^C). As shown in Supplementary Fig. 1A, we did not observe any major discrepancy between experimental and theoretical values, suggesting that under our experimental conditions, orbitrap-based mass spectrometer was not biased toward certain isotopologues. To rule out potential enrichment fraction bias introduced by natural abundance correction, we compared the ratio of M + 1 to M + 0 without natural abundance correction in cells treated with or without [U-^13^C]-glucose (Supplementary Fig. 1B). M + 1 to M + 0 ratio was significantly higher in samples receiving [U-^13^C]-glucose tracing than samples without tracing (^12^C-glucose) data, confirming the contribution of ^13^C tracer to the production of M + 1 species. Furthermore, Supplementary Fig. 2 suggests that [U-^13^C]-glutamine was close to 98%, so M + 1 labeling was not due to tracer impurity, either. A plausible explanation of M + 1 labeling is that decarboxylation of [U-^13^C]-glutamine produces ^13^CO_2_, which is subsequently fixed into the TCA cycle in vivo. This model challenges the common assumption that endogenous ^13^CO_2_ from decarboxylation of [U-^13^C]-glutamine is diluted by the body pool of bicarbonate, and thus, the contribution of ^13^CO_2_ to ^13^C-labeling is not significant. Furthermore, TCA reactions are often referred to as having a net flux in the clockwise direction. The observation of M + 1-labeled succinate, fumarate, and malate indicates the reverse reactions and exchange fluxes from oxaloacetate to succinate.

### M + 1-labeled TCA metabolites by [U-^13^C]-glucose is dominant in the in vivo tumor but not in cultured cells

**Fig. 2:**
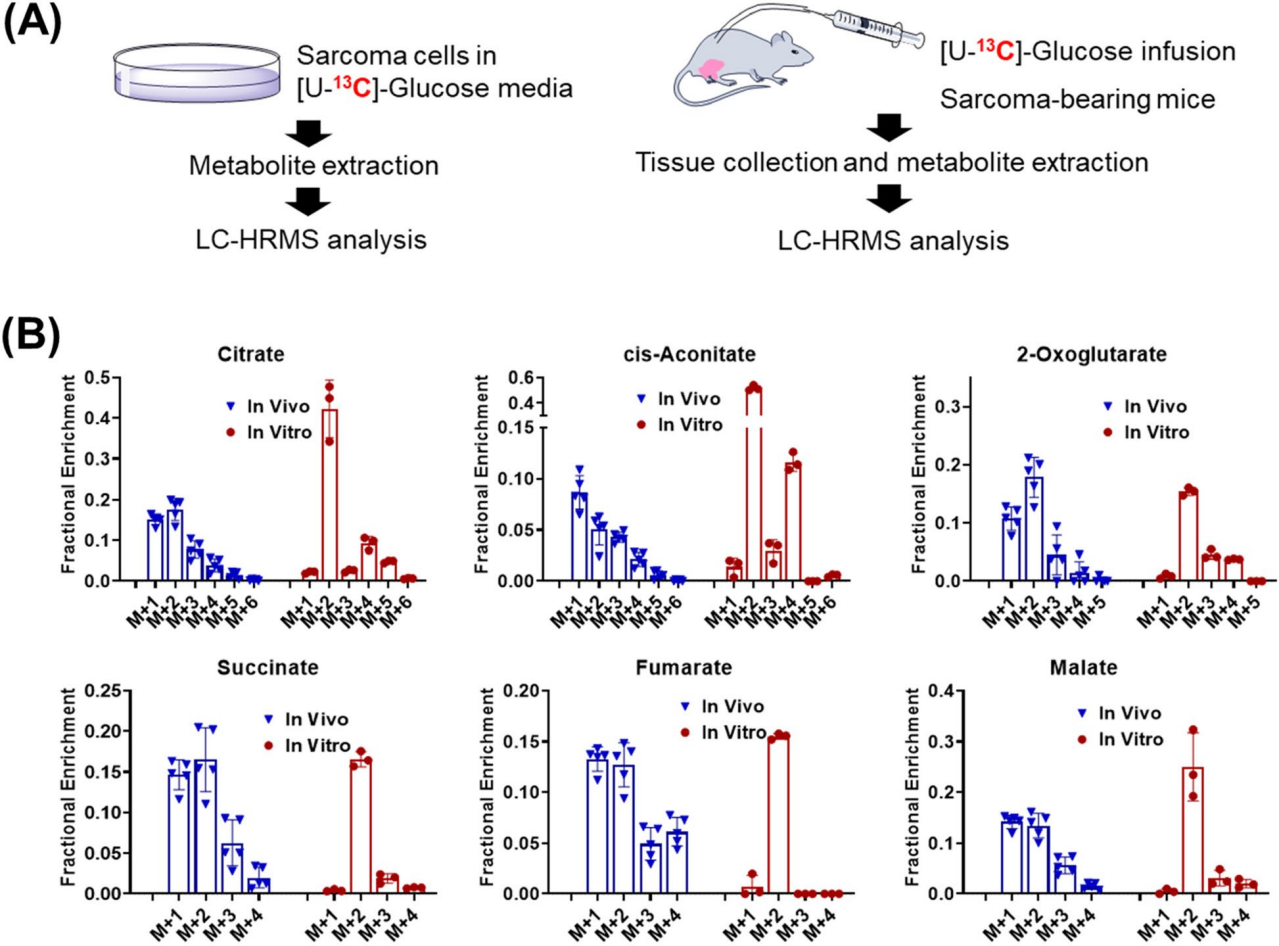
Distinct ^13^C labeling of TCA intermediates with [U-^13^C]-glucose is observed in in vitro cell cultures and in tumors in vivo. **A** Schematic of in vivo and in vitro tracing assays. **B** ^13^C labeling patterns of metabolites from sarcoma cells (in vitro) fed with [U-^13^C]-glucose medium for 3 h (h) and sarcoma tissue from sarcoma-bearing mice (in vivo) receiving [U-^13^C]-glucose infusion for 3 h. Data are presented as mean ± SD *n* = 5 for animals and *n* = 3 for cultured cells. Data in this figure was after natural abundance correction

To investigate whether glucose-derived CO_2_ can also be recycled, we next performed in vitro and in vivo tracing experiments using [U-^13^C]-glucose (Fig. 2A). Through glycolysis, [U-^13^C]-glucose is converted into [^13^C_3_]-pyruvate (M + 3), which then feeds a series of reactions in the TCA cycle. Pyruvate M + 3 is first converted into acetyl-CoA, and the M + 2 acetyl group of acetyl-CoA is then transferred to the four-carbon oxaloacetate to form citrate M + 2 (Fig. 2B). The citrate M + 2 is then dehydrated by aconitase to give cis-aconitate M + 2, which subsequently goes through a series of chemical transformations to yield α-ketoglutarate M + 2, losing one carboxyl group as CO_2_. After oxidative decarboxylation of α-ketoglutarate M + 2, a four-carbon chain is generated as succinyl-CoA, which is further converted into succinate M + 2. The second carbon lost as CO_2_ is originated from the carbon of oxaloacetate. The succinate M + 2 remains labeled in the next two enzymatic reactions to provide malate M + 2. During the second round of the TCA cycle, oxaloacetate M + 2 reacts with acetyl-CoA M + 2 and produces citrate M + 4. Indeed, M + 4 was detected in cultured sarcoma cells (in vitro) and in sarcomas in mice (in vivo), and M + 4 was less abundant than M + 2. Surprisingly, M + 1 species were the dominant isotopologues for all the TCA metabolites in the tumor but not in the cultured cancer cells. Taken together, the appearance of high abundant M + 1 TCA intermediates in [U-^13^C]-glutamine (Fig. 1) and [U-^13^C]-glucose tracing (Fig. 2) experiments can be explained by the recycling of endogenous ^13^CO_2_. It is worth noting that the recycling of CO_2_ may be regulated by multiple enzymes, including but not limited to pyruvate carboxylase, malic enzyme activity, propionyl-CoA carboxylase, or others.

### M + 1-labeling of TCA metabolites is affected by exogenous NaHCO_3_ and CO_2_

**Fig. 3:**
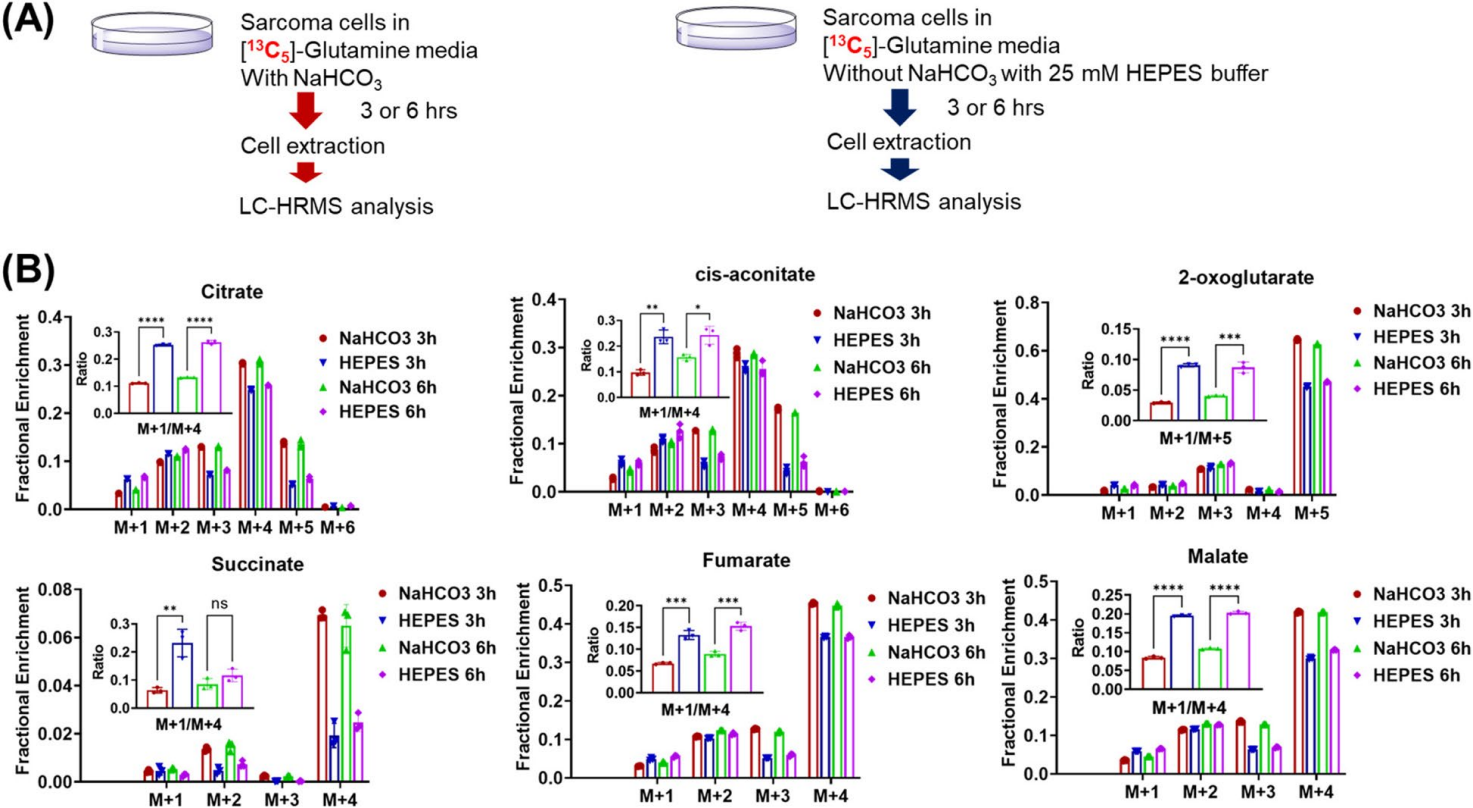
The effect of exogenous NaHCO_3_ on M + 1 labeling in sarcoma cells. **A** Schematic of in vitro tracing analysis in media containing NaHCO_3_ or HEPEPS buffer. **B** ^13^C labeling patterns of metabolites from sarcoma cells (in vitro) incubated in [U-^13^C]-glutamine medium for 3 or 6 h. M + 1/M + 4 ratio (in the insert) was used to account for the overall TCA activity perturbations due to medium changes. Data are presented as mean ± SD of three replicates and after natural abundance correction

If M + 1 is due to CO_2_ recycling, manipulation of exogenous NaHCO_3_ and CO_2_ is expected to affect M + 1 labeling. We then altered medium conditions by replacing NaHCO_3_ with HEPES or turning off CO_2_ tank in order to test the effect of exogenous NaHCO_3_ and CO_2_ on M + 1 labeling using sarcoma cell culture. To account for potential changes of TCA cycle activity due to medium conditions, M + 1/M + 4 ratio was also calculated to better reflect M + 1 labeling. As predicted, when NaHCO_3_ in RPMI 1640 medium was replaced with HEPES while 5% CO_2_ supply remained, M + 1 peak increased (Fig. 3). However, when both NaHCO_3_ and CO_2_ were left out (Supplementary Fig. 3), M + 1 peak decreased. The decrease of M + 1 in the absence of 5% CO_2_ may be because decreased CO_2_ partial pressure (from 5 to 0.04%) in air above medium led to a decreased CO_2_ solubility in medium and, hence, a faster release of endogenous CO_2_ to air. Altogether, these results suggest the role of exogenous NaHCO_3_ and CO_2_ on M + 1 labeling.

### M + 1-labeled TCA intermediates are also observed in non-tumor tissues in vivo

**Fig. 4:**
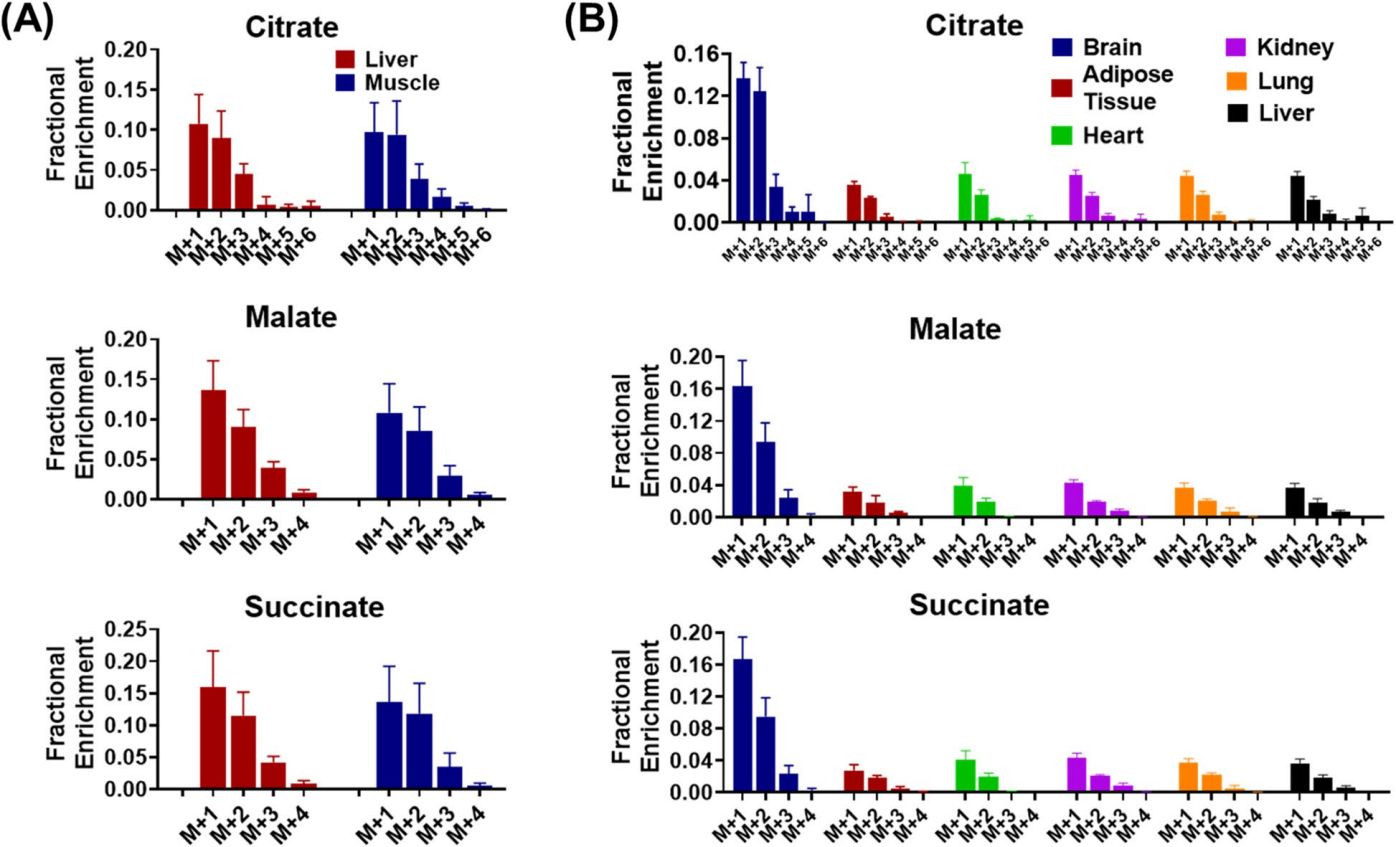
M + 1-labeled TCA intermediates are observed in non-tumor tissues in vivo. **A** ^13^C labeling patterns of metabolites in skeletal muscle and liver from sarcoma-bearing mice receiving [U-^13^C]-glucose infusion for 3 h. **B** ^13^C labeling patterns of metabolites in different types of organs (data from Hui et al., Nature 2017).^14^. Data are presented as mean ± SD *n* = 5 animals per group. Data in this figure was after natural abundance correction

To investigate whether CO_2_ recycling is a tumor-specific metabolic event or not, we further looked into the labeling patterns of TCA intermediates in non-tumor tissues. Similar to what we observed in tumor, citrate M + 1, succinate M + 1, and malate M + 1 were dominant isotopologues in mouse liver and skeletal muscle (Fig. 4A). In fact, a similar result of TCA intermediate enrichment in normal tissues after infusing mice with [U-^13^C]-glucose was reported by other independent investigators [14], but the source of the M + 1 species was not identified. We downloaded the relevant source data from the Supplementary material of this paper and analyzed the labeling pattern of citrate, succinate, and malate in different normal tissues (Fig. 4B). For citrate, the enrichment fractions of M + 1 and M + 2 were comparable. For succinate and malate, M + 1 was even more abundant than M + 2. These results indicate that endogenously generated ^13^CO_2_ participates in anaplerotic metabolism in non-tumor tissues as well.

### Endogenous CO_2_ contributes to purine biosynthesis in vivo

**Fig. 5:**
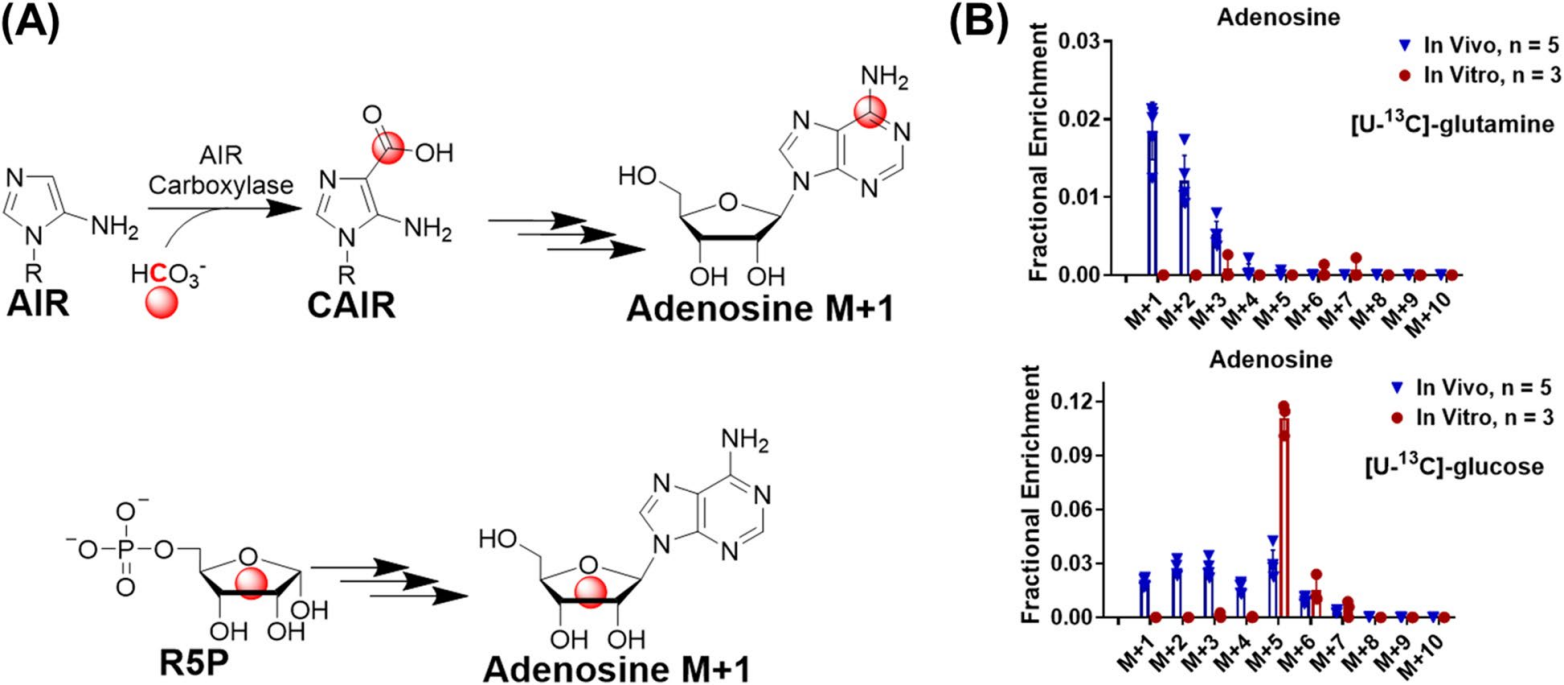
^13^CO_2_ contributes to adenosine biosynthesis. **A** Schematic of adenosine biosynthesis from various carbon sources. **B** ^13^C labeling pattern of adenosine from sarcoma cells fed with [U-^13^C]-glutamine/[U-^13^C]-glucose in vitro for 3 h and labeling patterns of adenosine in sarcomas from sarcoma-bearing mice receiving [U-^13^C]-glutamine/[U-^13^C]-glucose infusion in vivo for 3 h. Data are presented as mean ± SD *n* = 5 for animals and *n* = 3 for cultured cells. R5P, ribose 5-phosphate; AIR, aminoimidazole ribotide; CAIR, 5-amino-1-(5-phospho-D-ribosyl)imidazole-4-carboxylate. Data in this figure was after natural abundance correction

To explore whether CO_2_ recycling participates in other metabolic events, we then looked into de novo purine synthesis, which consumes CO_2_ (Fig. 5A). As shown in Supplementary Fig. 1B, we first confirmed that there was a significant increase of M + 1 adenosine in ^13^C tracing samples compared to samples without ^13^C tracing (“^12^C-glucose”). ^13^C labeling patterns of adenosine from sarcoma cells fed with [U-^13^C]-glutamine/[U-^13^C]-glucose in vitro and from sarcomas of sarcoma-bearing mice receiving [U-^13^C]-glutamine/[U-^13^C]-glucose infusion in vivo for 3 h are shown in Fig. 5B. No substantial labeling of adenosine with [U-^13^C]-glutamine was observed in cultured cells. However, adenosine M + 1 was detected in sarcomas from sarcoma-bearing mice receiving the [U-^13^C]-glutamine infusion, strongly suggesting that ^13^CO_2_ produced from [U-^13^C]-glutamine participates in purine biosynthesis. The [U-^13^C]-glucose tracing led to M + 5-labeled adenosine in both cultured cells and tumor, indicating the successful labeling of ribose 5-phosphate (R5P) by [U-^13^C]-glucose through the pentose phosphate pathway in vitro and in vivo. However, M + 1-labeling of adenosine with [U-^13^C]-glucose was only observed in the tumor in vivo. The observation of adenosine M + 1 in sarcomas after [U-^13^C]-glutamine or [U-^13^C]-glucose infusion in mice but not in cultured sarcoma cells is consistent with our previous observations that ^13^CO_2_ recycling is substantial in vivo but not in vitro (Figs. 1 and 2). Adenosine M + 1 can be labeled by ^13^CO_2_ in two ways:

1. An intermediate in purine biosynthesis, aminoimidazole ribonucleotide (AIR), is combined with ^13^CO_2_ by AIR carboxylase to produce CAIR M+1, which is subsequently converted into adenosine M+1 by adenosine aminohydrolase
2. ^13^CO_2_-labeled TCA intermediates (e.g., oxaloacetate or malate) can be used for glucose production (gluconeogenesis) and, hence, produce M + 1 glycolysis intermediates, which then contribute to ribose M + 1 in adenosine through labeling ribose 5-phosphate.

Here, we focus on possible routes for CO_2_ recycling, but the possibility also exists that M + 1 species in the gluconeogenic pathway are produced without ^13^CO_2_ recycling (e.g., production of M + 1 phosphoenolpyruvate via decarboxylation of [3,4-^13^C] oxaloacetate). In addition, non-oxidative pentose phosphate pathway may contribute to the production of M + 1 ribose-5 phosphate and glyceraldehyde 3-phosphate, leading to M + 1 labeling of downstream metabolites.

The location of ^13^C in adenosine will help to distinguish these two pathways, and we then collected the MS2 spectra of adenosine (Supplementary Fig. 4). The characteristic fragmentation of adenosine is the cleavage of base and glycosidic bond, resulting adenine and ribose fragments. Since both adenine and ribose contain equal number of carbons, then it is expected to observe a close to 1:1 ratio of ^12^C and ^13^C adenine in samples without ^13^C tracers. We set the precursor isolation window at 1 (*m/z*), and hence, M + 1 adenosine precursors may contain ^13^C adenosine and ^15^ N-adenosine. ^15^ N-adenosine will result in ^15^ N-adenine, and hence, we set the resolution of MS2 at 60,000 to distinguish ^13^C and ^15^ N adenine, so that ^15^ N-adenosine will not interfere with ^13^C-adenosine fragmentation patterns. Regarding the precursor ion ^13^C-adenosine, if ^13^C is mainly located in the adenine moiety of adenosine, then it is expected to observe around 2% higher ^13^C adenine in ^13^C tracing samples than in samples without ^13^C tracers, and, vice versa, if ^13^C is mainly located in the ribose moiety, then it is expected to see 2% higher ^12^C adenine in ^13^C tracing samples than samples without ^13^C tracers. Supplementary Fig. 4 demonstrates the adenosine M + 1 MS2 spectrum containing the characteristic fragments ^12^C and ^13^C adenine. There was no significant difference of adenine M + 1 relative abundance between samples with or without ^13^C tracers, suggesting that ^13^C in ^13^C-adenosine may reside in both adenine and ribose moiety. Separately, it is an interesting observation that in non-tracing samples (^12^C-glucose), adenine M + 1 was slightly lower than M + 0, and it is out of the scope of this study to identify the exact cause.

### Endogenous CO_2_ contributes to serine biosynthesis in vivo

**Fig. 6:**
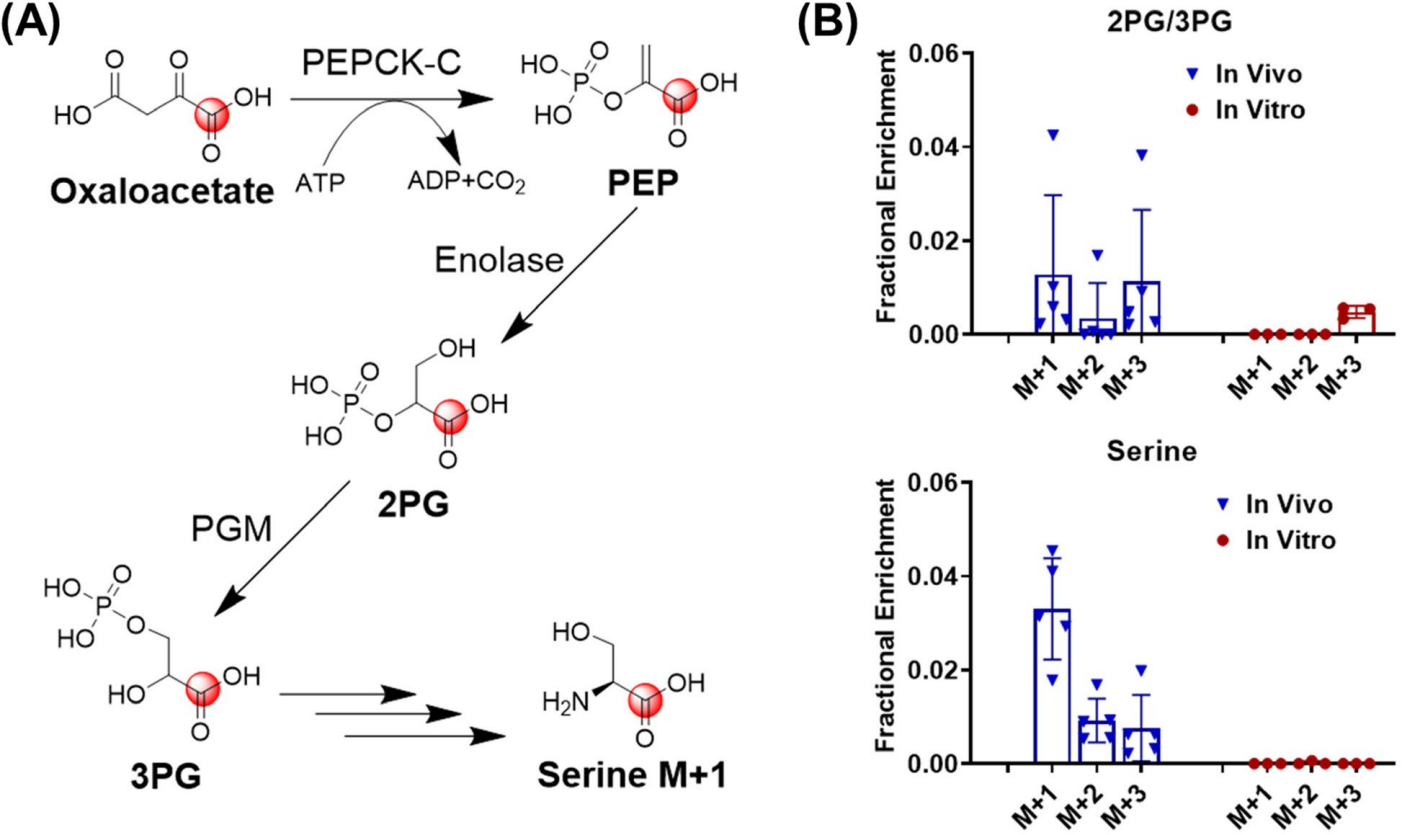
^13^CO_2_ contributes to serine biosynthesis. **A** Schematic of ^13^C incorporation into serine via the serine biosynthesis pathway. **B** ^13^C labeling pattern of 2PG/3PG and serine from sarcoma cells fed with [U-^13^C]-glutamine in vitro for 3 h and labeling patterns of 2PG/3PG and serine in sarcomas from sarcoma-bearing mice receiving [U-^13^C]-glutamine infusion in vivo for 3 h. Data are presented as mean ± SD *n* = 5 for animals and *n* = 3 for cultured cells. PEP, phosphoenolpyruvate; PEPCK-C, phosphoenolpyruvate carboxykinase; 2PG, 2-phosphoglyceric acid; 3PG, 3-phosphoglyceric acid; PGM, 2,3-bisphosphoglycerate-dependent phosphoglycerate mutase. Data in this figure was after natural abundance correction

We further investigated other ^13^C labeling patterns which can be explained by ^13^CO_2_ recycling. In the [U-^13^C]-glutamine tracing experiment, serine M + 1 was observed in mouse sarcomas in vivo but not in sarcoma cells cultured in a CO_2_ incubator (Fig. 6). Oxaloacetate M + 1 is generated by incorporating the ^13^CO_2_ with nonlabelled pyruvate (Fig. 6A). Oxaloacetate M + 1 is then converted into PEP M + 1 by introducing a phosphate group from ATP, catalyzed by PEPCK-C. PEP M + 1 is then hydrolyzed by enolase to produce 2PG M + 1, which is in equilibrium with 3PG M + 1 in the presence of PGAM. 3PG M + 1 is eventually converted into serine M + 1. Indeed, consistent with the model that ^13^CO_2_ recycling is substantial in vivo, 2PG/3PG M + 1 was only observed in tumors after in vivo infusion, but not in cultured tumor cells (Fig. 6B). Subsequently, de novo serine biosynthesis from 3PG M + 1 leads to the production of serine M + 1 (Fig. 6B). These results suggest that ^13^CO_2_ participates in the biosynthesis of serine in vivo.

### Citrate M + 1 appears earlier than M + 3 in vivo

**Fig. 7:**
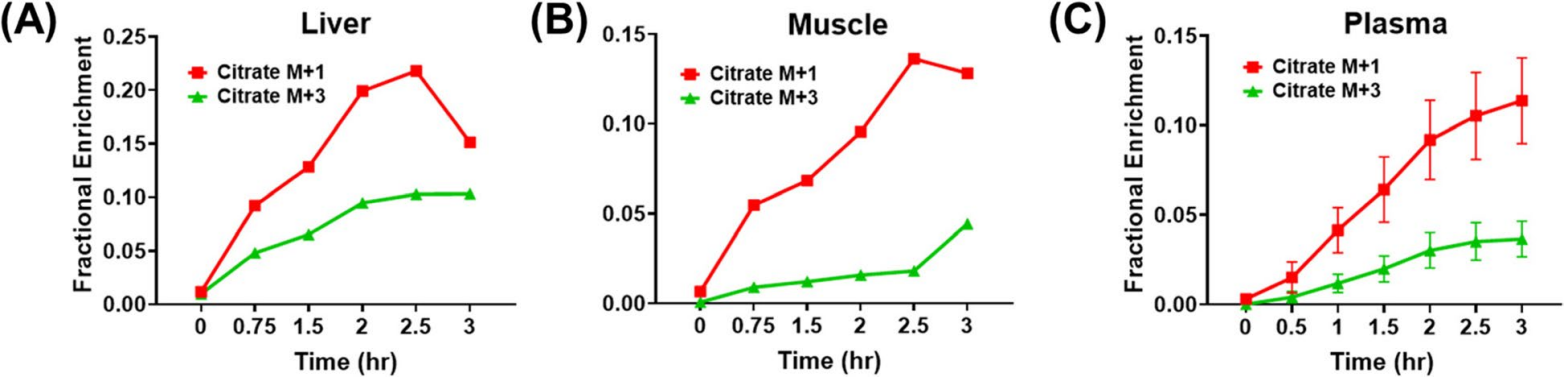
Plasma citrate M + 1 appears earlier than M + 3. Time course of citrate M + 1 and M + 3 in the liver (**A**), skeletal muscle (**B**), and plasma (**C**) of mice receiving [U-^13^C]-glucose infusion for 3 h (h). For data in **A** and **B**, *n* = 1. Data in **C** are presented as mean ± SD *n* = 5 animals per group for the plasma sample only. Data in this figure was after natural abundance correction

In the [U-^13^C]-glucose tracing experiment, [^13^C_3_]-pyruvate (M + 3) is produced through glycolysis. Pyruvate M + 3 enters the TCA cycle through pyruvate dehydrogenase or pyruvate carboxylase, leading to the production of M + 2 acetyl-CoA or M + 3 oxaloacetate, respectively, which subsequently contributes to the production of citrate M + 2 and M + 3, respectively. Hence, citrate M + 3 is usually used as a marker of pyruvate carboxylase in many studies [1]. Surprisingly, the time-course experiment (Fig. 7) demonstrated that citrate M + 1 was more abundant than M + 3 and also appeared earlier than M + 3 in the liver, skeletal muscle, and plasma of mice receiving [U-^13^C]-glucose tracing. These results suggest that HCO_3_^−^ pool may be labeled more rapidly than pyruvate. ^13^C-HCO_3_^−^ and unlabeled pyruvate can then lead to M + 1 labeling, and this also potentially explains why citrate M + 4 was less abundant than M1 in sarcoma of mice treated with [U-^13^C]-glucose (Fig. 2).

**Fig. 8:**
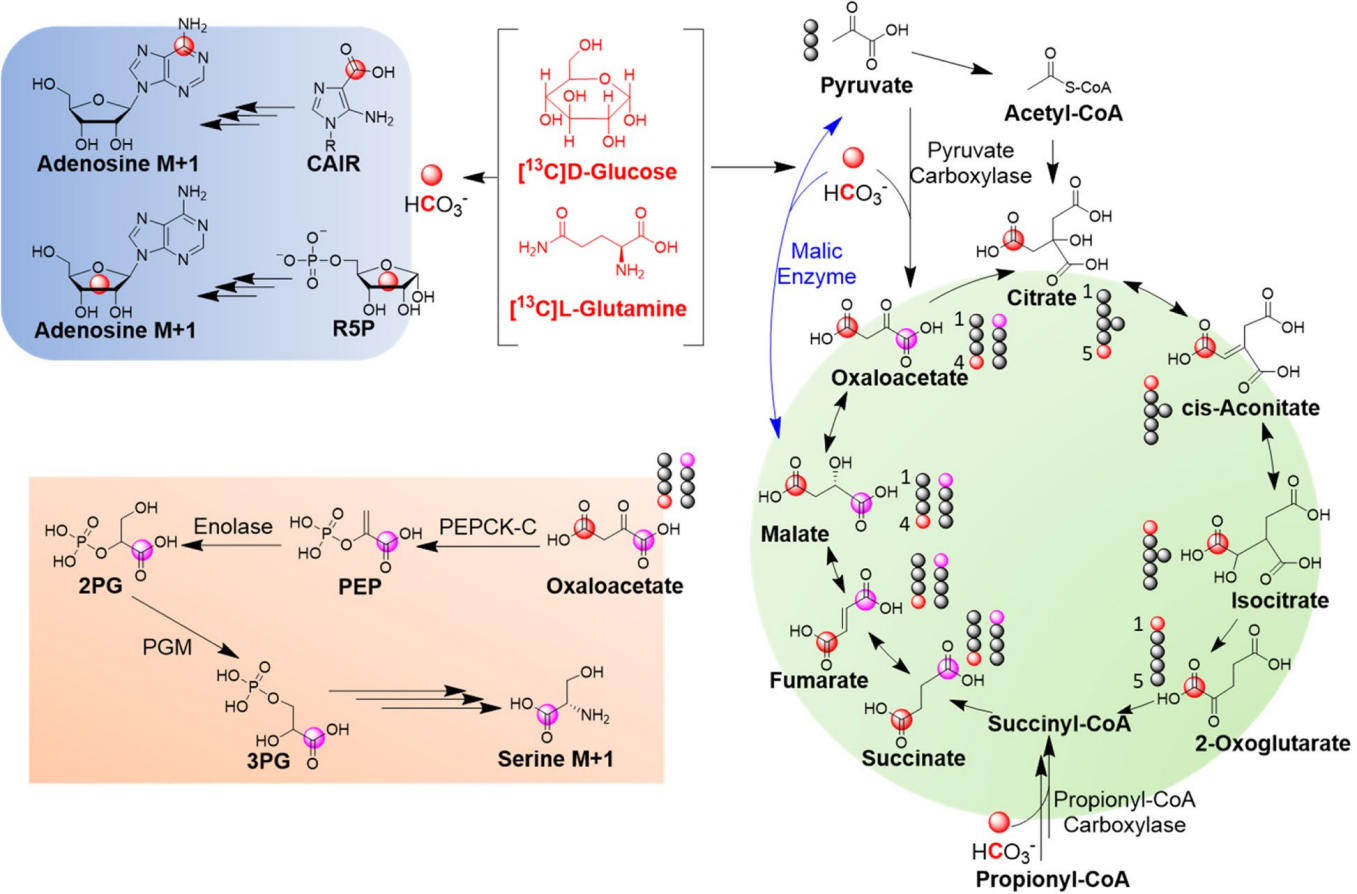
Proposed routes of CO_2_ incorporation into different metabolomic pathways. [4-^13^C]-Oxaloacetate produced from pyruvate carboxylation with ^13^CO_2_ is assumed to result in [1-^13^C] and [4-^13^C]-fumarate, since fumarate is a symmetrical molecule and malate dehydrogenase and fumarase catalyze reversible reactions. Red and pink balls represent ^13^C. Pink ball illustrates the carbon scrambling in fumarate

When taken together, these results suggest that even though ^13^CO_2_ produced from the decarboxylation of ^13^C tracers is negligible in vitro, endogenous ^13^CO_2_ produced in vivo is substantial and subsequently fixed into the TCA cycle, glycolysis intermediates, ribose, purine, and serine, resulting in M + 1 isotopologues in different metabolic pathways, which are summarized in Fig. 8.

## Discussion

Compared to metabolomics which detects changes in metabolite levels, stable isotope tracing is more informative, because it provides activity readout of metabolic enzymes in intact cells or whole organisms. Precisely relating metabolite enrichment patterns to a specific metabolic enzyme or metabolic pathway requires in-depth knowledge of metabolic reactions and biological systems in which the isotope tracing assay is performed. We performed [U-^13^C]-glutamine and [U-^13^C]-glucose tracing in sarcoma cells cultured in standard in vitro cell culture conditions and also in vivo in mice. We observed distinct enrichment patterns of TCA intermediates, featured by high-abundant M + 1 species in mice, but not in cultured cells. Interestingly, observing high-abundant M + 1 species in vivo recapitulates previously published results by other groups [8, 11, 14, 15], but the reason for the difference in the M + 1 species between in vivo and in vitro experiments has not previously been explained. Our results are consistent with a model where the difference in M + 1 species is due to the different sources of CO_2_ in vivo and in vitro. Endogenous CO_2_ in cell culture is diluted by exogenous CO_2_ and NaHCO_3_ from the artificial conditions within an incubator, and hence, the ^13^C enrichment by [U-^13^C]-glutamine or [U-^13^C]-glucose is negligible. In contrast, CO_2_ from endogenous metabolism is substantial in mice, and our results indicate that CO_2_ is labeled in the presence of [U-^13^C]-glucose or [U-^13^C]-glutamine. ^13^CO_2_ reenters the TCA cycle via pyruvate carboxylase-mediated pyruvate carboxylation, resulting in M + 1-labeled TCA intermediates. High-abundant M + 1 TCA species in multiple tissues of mice receiving [U-^13^C]-glucose were also observed by other groups. [U-^13^C]-glutamine may contribute to ^13^CO_2_ production via α-ketoglutarate dehydrogenase- and isocitrate dehydrogenase-mediated decarboxylation reactions. [U-^13^C]-glucose may contribute to ^13^CO_2_ production via oxidative pentose phosphate pathway or pyruvate dehydrogenase-mediated pyruvate decarboxylation. These findings suggest that special attention needs to be paid when M + 1 species are used to track specific pathways. For example, [1-^13^C]-glutamine can be used as a tracer in cancer cells where citrate M + 1 is used to monitor reductive glutaminolysis activity. This approach is acceptable when it is used in vitro because the endogenous CO_2_ contribution is negligible in the presence of an exogenous CO_2_ supplement. However, our findings reveal that this approach should be used with caution to estimate reductive glutaminolysis in vivo, because [1-^13^C]-glutamine will label CO_2_, and citrate M + 1 may come from pyruvate carboxylation and/or reductive glutaminolysis. In addition, adenosine M + 1 may be misinterpreted as the contribution via one-carbon metabolism in vivo, but [U-^13^C]-glutamine is not likely contributing to one-carbon metabolism, so adenosine M + 1 is also due to recycling ^13^CO_2_.

It is noteworthy that other metabolic pathways (e.g., propionyl-CoA carboxylation, malic enzyme) may also contribute to M + 1 labeling (Fig. 8). For example, if all TCA reactions are clockwise unidirectional, then ^13^C from ^13^C CO_2_ may enter TCA cycle via oxaloacetate and remain in TCA intermediates through alpha-ketoglutarate and then be lost in the next step, resulting in unlabeled succinyl-CoA. To test the possibility, we performed acyl-CoA profiling and plotted ^13^C labeling patterns of succinyl-CoA in sarcoma of mice treated with [U-^13^C]-glucose (Supplementary Fig. 5). Our experimental results suggest that succinyl-CoA shared identical labeling patterns as succinate, and there are two possibilities to explain this result: (1) TCA reactions at certain steps have reversibility and lead to isotopic exchange, and (2) propionyl-CoA carboxylation using ^13^C CO_2_ leads to M + 1 labeling.

First, based on standard Gibbs free energy change (ΔG0′), few reactions in TCA cycle are readily reversible or near-equilibrium reactions with a ΔG0′ close to zero. For example, the conversion of succinyl-CoA to succinate is catalyzed by succinyl-CoA synthetase, and this reaction has ΔG0′ of − 2.9 kJ/mol. Indeed, previous studies have already shown the reversibility of succinyl-CoA synthetase [16]. Similarly, ΔG0′ is 0.0 kJ/mol for succinate dehydrogenase and − 3.8 kJ/mol for fumarase. The reversibility of these two enzymes are supported by previous studies [17, 18]. In addition, mitochondrial malate dehydrogenase is typically considered a reversible enzyme as well [19]. Hence, oxaloacetate M + 1 can be converted to malate M + 1 via reverse reaction catalyzed by malate dehydrogenase, followed by fumarate M + 1 via reverse reaction catalyzed by fumarase. Similarly, fumarate M + 1 can lead to succinate M + 1 production, followed by the formation of succinyl-CoA M + 1 via reverse reactions. In fact, in response to the ignorance of the reversibility of several steps in TCA cycle, Krebs himself stated that the reactions succinate ↔ fumarate ↔ malate ↔ oxaloacetate are reversible [20].

Second, regarding propionyl-CoA carboxylation, in mice or human, propionyl-CoA may be derived from propionate, which is mainly produced by gut bacteria, or branched chain amino acids (BCAAs, e.g., valine, isoleucine), or methionine and threonine. Propionate produced in gut is delivered to the liver through portal vein, and the major part of propionate is processed by the liver [21], resulting in low concentration (low micromolar range) of propionate in blood [22]. One possibility is that ^13^C CO_2_ recycling via propionyl-CoA carboxylase may produce glucose M + 1 through gluconeogenesis in the liver, and then, glucose M + 1 may be released to blood and utilized by other tissues to produce M + 1 metabolites. In our study, plasma glucose M + 1 was detected (Supplementary Fig. 6).

Furthermore, BCAAs are readily available and can be rapidly oxidized by multiple tissues, but it was reported that in most tissues, BCAAs only supply 1–6% of carbons in the TCA cycle, while the pancreas can use BCAA carbons to supply 20% of the TCA carbons [23]. The fractional contribution of BCAA to the TCA in skeletal muscle was reported to be much smaller (5–6%) than that of fatty acids (40%) [23]. In our studies, we found that in mouse sarcoma, glucose and glutamine together contribute to 67% carbons in succinate (26% from glucose and 41% from glutamine based on ^13^C tracing data), suggesting that the contribution of other sources is relatively small. In another word, if M + 1 citrate is from propionyl-CoA carboxylase, then it is expected to see a lower citrate M + 1 than M + 2 in [U-^13^C]-glucose tracing and M + 4 in [U-^13^C]-glutamine labeling. However, we observed a higher M + 1 in [U-^13^C]-glucose tracing and [U-^13^C]-glutamine tracing (Figs. 1– 2), suggesting that propionyl-CoA carboxylation may not be the major source of M + 1 labeling. Nevertheless, a more quantitative study using models of varying pyruvate carboxylase and propionyl-CoA carboxylase activities will be needed to further evaluate their role in M + 1 labeling.

Besides pyruvate carboxylase and propionyl-CoA carboxylase, malic enzyme may also contribute to CO_2_ recycling by catalyzing the conversion of pyruvate and bicarbonate to malate [12]. In addition, in the [U-^13^C]-glutamine tracing assay, pyruvate M + 1 may be generated from malate M + 2 (^13^C at C3 and C4) via malic enzyme, and via the reverse reaction, malate M + 1 will be produced. Nevertheless, pyruvate M + 1 is only 4% (Supplementary Fig. 7), suggesting that malate to pyruvate conversion may not be dominant under our experimental conditions, while the contribution of malic enzyme to pyruvate carboxylation will need further investigation.

In this study, we primarily focus on possible routes for CO_2_ recycling, but it is important to mention that M + 1 species in the gluconeogenic pathway can also be produced without ^13^CO_2_ recycling (e.g., production of M + 1 PEP via decarboxylation of [3,4-^13^C] oxaloacetate). In addition, M + 1 ribose-5 phosphate and glyceraldehyde 3-phosphate may also be produced from pentose phosphate pathway, leading to M + 1 labeling of downstream metabolites, such as adenosine, serine, pyruvate, and TCA intermediates (Figs. 5, 6, 7 and 8). However, under our experimental conditions, the labeling percentage of M + 1 TCA intermediates was generally higher than M + 1 glycolysis intermediates in tissues (Figs. 1 and 6) and M + 1 pyruvate or glucose in plasma (Supplementary Figs. 6-7), suggesting the presence of other M + 1 labeling pathways, e.g., CO_2_ recycling.

Replenishing the TCA cycle (a process termed anaplerosis) is important to maintain homeostasis. The carbon source of anaplerosis varies from tissue to tissue and is also dependent on physiological conditions (e.g., fast vs fed state). Pyruvate carboxylase has been identified to play an important role in regulating anaplerosis [11]. Hence, pyruvate carboxylase is involved in various types of diseases and is an emerging therapeutic target. Pyruvate carboxylase deficiency is a genetic disease present at birth, leading to damage to tissues and organs. Pyruvate carboxylase is also involved in tumorigenesis [10, 24–26]. High pyruvate carboxylase was shown to be correlated with glycemia [27]. Tissue-specific inhibition of pyruvate carboxylase reduced plasma glucose concentrations and could be a potential therapeutic approach for nonalcoholic fatty liver disease, hepatic insulin resistance, and type 2 diabetes [27]. Citrate M + 3 is often used to reflect pyruvate carboxylase activity in [U-^13^C]-glucose tracing experiments, but our results show that citrate M + 1 is more abundant than citrate M + 3 in both tissues and plasma, and it would be of importance to determine whether M + 1 citrate in plasma is a potential readout of tissue pyruvate carboxylase or not. Additional experiments and animal models (e.g., pyruvate carboxylase-depleted mice) are warranted to evaluate the potential role of citrate M + 1 as a noninvasive marker of tissue pyruvate carboxylase activity.

Furthermore, CO_2_ recycling may also affect the interpretation of the respiratory quotient (RQ), which is the ratio of CO_2_ production to O_2_ consumption. For every mole of glucose undergoing complete oxidation, 6 mol of CO_2_ are produced and 6 mol of O_2_ are consumed, so the RQ is 1. Each mole of palmitate produces 16 mol of CO_2_ and consumes 23 mol of O_2_, so the RQ is close to 0.7 when palmitate is completely oxidized. Hence, the RQ is used as an indicator of energy fuel (e.g., carbohydrate or fat) being metabolized in the body and helps to plan nutritional therapy. The RQ is determined by comparing exhaled gases to room air, but this approach does not take in vivo CO_2_ recycling into consideration. Therefore, the RQ will be underestimated when CO_2_ recycling is substantial, resulting in an inaccurate interpretation of fuel source utilization.

## Conclusion

In summary, understanding enrichment patterns and individual isotopologue provides rich information on metabolic reactions. The production and recycling of ^13^CO_2_ from the decarboxylation of [U-^13^C]-glucose or [U-^13^C]-glutamine is negligible in vitro due to dilution by the exogenous HCO_3_^–^/CO_2_ source, but in in vivo, the incorporation of endogenous ^13^CO_2_ into M + 1 TCA intermediates and other metabolites is substantial and should be considered. M + 1 TCA intermediates in vivo also suggest the isotopic exchange of oxaloacetate, malate, fumarate, and succinate, indicating the reversibility of malate dehydrogenase, fumarase, and succinate dehydrogenase. These findings not only provide an interpretation of distinct labeling patterns of TCA intermediates in vivo and in vitro but also provide insights into the proper design of ^13^C tracing experiments and modeling when ^13^C tracers are used to study in vivo metabolism. The current data is not sufficient to determine whether plasma M + 1 citrate is a readout of tissue pyruvate carboxylase activity or not, and additional experiments are warranted.

### Experimental procedures

#### Reagents

Optima LC–MS grade of ammonium acetate, water, acetonitrile, and methanol was purchased from Fisher Scientific. [U-^13^C_6_]-glucose and [U-^13^C_6_]-glutamine were obtained from the Cambridge Isotope Laboratories. RPMI 1640 medium and fetal bovine serum (FBS) were obtained from the Thermo Fisher Scientific. Dialyzed FBS was obtained from the Thermo Fisher Scientific. Jugular vein catheters, vascular access buttons, and infusion equipment were purchased from the Instech Laboratories.

#### Cell culture

Mouse primary sarcoma cell lines were generated from Pax7CreER-T2, p53^FL/FL^, and LSL-Nras^G12D^ tumors as previously described [28]. Mouse sarcoma-derived cell lines were cultured in a 10-cm dish with full growth medium containing RPMI 1640 supplemented with 10% FBS. The cell incubator was set at 37 °C supplemented with 5% CO_2_. Cells were then seeded into 6-well plates. After overnight incubation in the full growth medium, the old medium was replaced with 1.5 ml of RPMI 1640 (supplemented with 10% dialyzed FBS) containing 11.1 mM [U-^13^C]-glucose or 2 mM [U-^13^C]-glutamine. To study the effect of exogenous NaHCO_3_ and CO_2_ on M + 1 labeling in sarcoma cells, NaHCO_3_ was substituted with 20 mM HEPES buffer, and or the CO_2_ gas tank was disconnected from cell incubator so that the CO_2_ concentration in cell incubator was reduced from 5 to 0.04% (atmospheric CO_2_ concentration). ^13^C tracing started by replacing medium with glutamine-free medium supplemented with 2 mM [U-^13^C]-glutamine. After 3- or 6-h incubation of sarcoma cells in the presence of [U-^13^C]-glutamine, intracellular metabolites were harvested.

#### Animal models

All animal procedures were approved by the Institutional Animal Care and Use Committee (IACUC) at Duke University. The mouse model of soft-tissue sarcoma was generated on a mixed background (129/SvJae and C57BL/6) using a combination of alleles that were previously described: Pax7^CreER−T2^ [29], p53^FL/FL^ [30], LSL-Nras^G12D^ [31], and ROSA26^mTmG^. Primary mouse soft tissue sarcomas were generated in the mouse hind limb as previously described [28] by intramuscular (IM) injection of (Z)-4-hydroxytamoxifen (4-OHT). 4-OHT was dissolved in 100% DMSO at a concentration of 10 mg/ml, and 50 μl of the solution was injected into the gastrocnemius muscle.

#### In vivo ^13^C glucose and glutamine infusions

To perform in vivo nutrient infusions, chronic indwelling catheters were placed into the right jugular veins of mice, and animals were allowed to recover for 3–4 days prior to infusions. Mice were infused with [U-^13^C]-glucose for 3 h at a rate of 20 mg/kg/min (150 µl/h). Blood was collected via the tail vein at 0, 30 min, 1, 1.5, 2, 2.5, and 3 h. The plasma was collected by centrifuging blood at 3000 g for 15 min at 4 °C. At the end of infusions, tissues were snap-frozen in liquid nitrogen and stored at − 80 °C for further analyses. [U-^13^C]-glutamine (Cambridge Isotope Laboratories) was infused for 3 h at a rate of 6 mg/kg/min (200 µl/h).

#### HPLC method

The analysis of metabolites in mouse tissues and plasma was performed using Ultimate 3000 UHPLC (Dionex), while the analysis of metabolites from cultured cells was performed using Vanquish UHPLC (Thermo Fisher Scientific). A hydrophilic interaction chromatography method (HILIC) with an XBridge Amide column (100 × 2.1 mm i.d., 3.5 μm; waters) was used for compound separation at 25 °C: mobile phase A: water with 5 mM ammonium acetate (pH 6.8) and mobile phase B: 100% acetonitrile. Linear gradient is as follows: 0 min, 85% B; 1.5 min, 85% B; 5.5 min, 35% B; 6.9 min, 35% B; 10.5 min, 35% B; 10.6 min, 10% B; 12.5 min, 10% B; 13.5 min, 85% B; 17.9 min, 85% B; 18 min, 85% B; and 20 min, 85% B. Due to the instrumentation difference between Ultimate 3000 UHPLC and Vanquish UHPLC, different flow rates were used. For Ultimate 3000 UHPLC, the flow rate is as follows: 0–5.5 min, 0.15 ml/min; 6.9–10.5 min, 0.17 ml/min; 10.6–17.9 min, 0.3 ml/min; and 18–20 min, 0.15 ml/min. For Vanquish UHPLC, the flow rate is as follows: 0–5.5 min, 0.11 ml/min; 6.9–10.5 min, 0.13 ml/min; 10.6–17.9 min, 0.25 ml/min; and 18–20 min, 0.11 ml/min.

#### Mass spectrometry

The analysis of metabolites in mouse tissues and plasma was performed on Q Exactive Plus mass spectrometer (Thermo Fisher Scientific), while the analysis of metabolites from cultured cells was performed on Orbitrap Exploris 480 mass spectrometer (Thermo Fisher Scientific). Both mass spectrometers were equipped with a HESI probe and operated in the positive/negative switching mode. When Q Exactive Plus mass spectrometer was used, the relevant parameters are as listed: heater temperature, 120 °C; sheath gas, 30; auxiliary gas, 10; sweep gas, 3; spray voltage, 3.6 kV for positive mode and 2.5 kV for negative mode; capillary temperature, 320 °C; and S-lens, 55. The resolution was set at 70,000 (at m/z 200). Maximum injection time (max IT) was set at 200 ms, and automatic gain control (AGC) was set at 3 × 10^6^. When Exploris 480 mass spectrometer was used, the relevant parameters are as listed: vaporizer temperature, 350 °C; ion transfer tube temperature, 300 °C; sheath gas, 35; auxiliary gas, 7; sweep gas, 1; spray voltage, 3.5 kV for positive mode and 2.5 kV for negative mode; and RF lens (%), 30. The resolution was set at 60,000 (at m/z 200). Automatic maximum injection time (max IT) and automatic gain control (AGC) were used. To collect MS2 spectra of adenosine isotopologues, Orbitrap Exploris 480 was operated in targeted MS2 mode choosing the following precursor ions in positive ion mode: adenosine M + 0 (m/z 226.0895) and adenosine M + 1 (m/z 267.0929). The MS2 condition was set as follows: precursor isolation window was set at 1 (m/z), HCD collision energy was set at 30%, and orbitrap resolution was set at 60,000. Succinyl-CoA analysis was carried out using Q Exactive Plus mass spectrometer (Thermo Fisher Scientific) following a condition described in a previously published article [32].

#### Metabolite extraction from cultured cells, tissues, and plasma

To harvest intracellular metabolites, cells were briefly washed with ice-cold saline (0.9% NaCl, 1 ml, twice) and immediately placed on dry ice before they were extracted into 1 ml extraction solution composed of 80% methanol/water (pre-cooled in − 80 °C freezer). Samples were centrifuged at 20,000 g for 10 min at 4 °C, and the supernatant was split into two Eppendorf tubes before drying in a speed vacuum concentrator (Labconco). The dry pellets were reconstituted into 30 μl sample solvent (water:methanol:acetonitrile, 2:1:1, v/v), and 3 μl was injected into the LC-HRMS. The tumor sample was first homogenized in liquid nitrogen, and then, 5 to 10 mg was weighed into a new Eppendorf tube. Ice-cold extraction solvent (250 μl) was added to the tissue sample, and a pellet mixer was used to further break down the tissue chunk and form an even suspension, followed by the addition of 250 μl to rinse the pellet mixer. After incubation on ice for an additional 10 min, the tissue extract was centrifuged with a speed of 20,000 g at 4 °C for 10 min. The dry pellets were reconstituted into 30 μl (per 3 mg tissue) sample solvent (water:methanol: acetonitrile, 2:1:1, v/v), and 3 μl was injected to LC-HRMS. A total of 5 μl mouse plasma was mixed with 5 μl water, and 40 μl ice-cold methanol was added. After vortex for 1 min, the mixture was centrifuged with a speed of 20,000 g at 4 °C for 10 min, and 3 μl was injected to LC-HRMS. To harvest succinyl-CoA, around 60 mg tumor from each mouse was weighed in a new Eppendorf tube, followed by extraction using ice-cold extraction solvent (water:methanol, 1:4, v/v, 500 μl). After centrifugation at 20,000 g at 4 °C for 10 min, the supernatant containing around 15 mg tumor was aliquoted and then dried using speed vacuum evaporator. The dry pellets were reconstituted into 30 μl sample solvent (50 mM ammonium acetate), and 10 μl was injected to LC-HRMS for further analysis.

#### Data analysis and statistics

LC–MS peak extraction and integration were performed using commercially available software Sieve 2.0 (Thermo Fisher Scientific). The integrated peak area was used to calculate ^13^C enrichment. Natural abundance correction was performed using software R with Bioconductor R package IsoCorrectoR [33]. All data were represented as mean ± SD. All *p*-values were obtained from the Student’s *t*-test (two-tailed) using GraphPad Prism 8 unless otherwise noted.

## Supplementary Information

**Additional file 1: SupplementaryFigure 1. **The comparison ofexperimental and theoretical values of M+1 relative abundance of representativemetabolites analyzed by orbitrap-based mass spectrometer. (A) The experimental and theoretical values of M+1 abundance ofrepresentative metabolites. (B) The abundance of adenosine M+1 (relative toM+0) in cells treated with or without [U-^13^C]-glucose. The relativeratio of M+0 is set as 1 and M+1 abundance is normalized to M+0 abundance. Thedata presented in this figure was without natural abundance correction. **SupplementaryFigure 2.** The mass isotopologue distribution of [U-^13^C]-glutaminedetected in glutamine free RPMI 1640 medium supplemented with [U-^13^C]-glutamine. The data presented in this figure waswithout natural abundance correction. **SupplementaryFigure 3.** The effect of exogenous CO_2_ on M+1 labeling in sarcomacells. (A) Schematic of in vitrotracing analysis. (B) ^13^C labeling patterns of metabolites fromsarcoma cells (in vitro) incubated in [U-^13^C]-glutamine medium inthe presence or absence of CO_2_ for 3 hrs. M+1/M+4 ratio (in theinsert) was used to account for the overall TCA activity perturbations due tomedium changes. Data are presented as mean ± SD of three replicates and afternatural abundance correction. **Supplementary Figure 4.** MS2 analysis of adenosine M+1. (A) TheMS2 spectrum of adenosine fragments. (B) the relative ratio of daughter ionadenine M+0 versus adenine M+1 in the absence of tracer (labeled as “^12^C-glucose”)or [U-^13^C]-glucose. Data in (B) are presented as mean ± SD of threereplicates. P value was calculated based on Student’s ttest, and “ns” denotes p value larger than 0.05. The data presented in thisfigure was without natural abundance correction. **Supplementary Figure 5.** The mass isotopologue distribution of succinyl-CoA in sarcomaof mice receiving [U-^13^C]-glucose tracing. Data are presented asmean ± SD of n=5 mice. Data with and without natural abundance (NA) correctionwas presented. **Supplementary Figure 6.** GlucoseM+1 in the plasma of mice receiving [U-^13^C]-glucose for 3 hrs. Datapresented here was after natural abundance correction. **Supplementary Figure 7.** Pyruvate labeling patterns in sarcoma of micereceiving [U-^13^C]-glucose or [U-^13^C]-glutamine tracingfor 3 hrs. Data are presented as mean ± SD of three replicates and afternatural abundance correction.

## Data Availability

The data is available upon request from the corresponding author.

## Abbreviations

LC: Liquid chromatography
HRMS: High-resolution mass spectrometry
TCA cycle: Tricarboxylic acid cycle
R5P: Ribose 5-phosphate
AIR: Aminoimidazole ribotide
CAIR: 5-Amino-1-(5-phospho-D-ribosyl)imidazole-4-carboxylate
PEP: Phosphoenolpyruvate
PEPCK-C: Phosphoenolpyruvate carboxykinase
2PG: 2-Phosphoglyceric acid
3PG: 3-Phosphoglyceric acid
PGM: 2,3-Bisphosphoglycerate-dependent phosphoglycerate mutase
BCAAs: Branched chain amino acids
