# Supplementary material for: 13C tracer analysis suggests extensive recycling of endogenous CO2 in vivo
Source: Cancer Metab. 2022 Jul 7;10:11. doi: 10.1186/s40170-022-00287-8 (PMC9264524; doi:10.1186/s40170-022-00287-8)
Supplement: Supplementary file 1 — Additional file 1: SupplementaryFigure 1. The comparison ofexperimental and theoretical values of M+1 relative abundance of representativemetabolites analyzed by orbitrap-based mass spectrometer. (A) The experimental and theoretical values of M+1 abundance ofrepresentative metabolites. (B) The abundance of adenosine M+1 (relative toM+0) in cells treated with or without [U-13C]-glucose. The relativeratio of M+0 is set as 1 and M+1 abundance is normalized to M+0 abundance. Thedata presented in this figure was without natural abundance correction. SupplementaryFigure 2. The mass isotopologue distribution of [U-13C]-glutaminedetected in glutamine free RPMI 1640 medium supplemented with [U-13C]-glutamine. The data presented in this figure waswithout natural abundance correction. SupplementaryFigure 3. The effect of exogenous CO2 on M+1 labeling in sarcomacells. (A) Schematic of in vitrotracing analysis. (B) 13C labeling patterns of metabolites fromsarcoma cells (in vitro) incubated in [U-13C]-glutamine medium inthe presence or absence of CO2 for 3 hrs. M+1/M+4 ratio (in theinsert) was used to account for the overall TCA activity perturbations due tomedium changes. Data are presented as mean ± SD of three replicates and afternatural abundance correction. Supplementary Figure 4. MS2 analysis of adenosine M+1. (A) TheMS2 spectrum of adenosine fragments. (B) the relative ratio of daughter ionadenine M+0 versus adenine M+1 in the absence of tracer (labeled as “12C-glucose”)or [U-13C]-glucose. Data in (B) are presented as mean ± SD of threereplicates. P value was calculated based on Student’s ttest, and “ns” denotes p value larger than 0.05. The data presented in thisfigure was without natural abundance correction. Supplementary Figure 5. The mass isotopologue distribution of succinyl-CoA in sarcomaof mice receiving [U-13C]-glucose tracing. Data are presented asmean ± SD of n=5 mice. Data with and without natural abundance (NA) correctionwas presented. Supplementary Figure 6. [file 40170_2022_287_MOESM1_ESM.docx]

**Additional file 1**


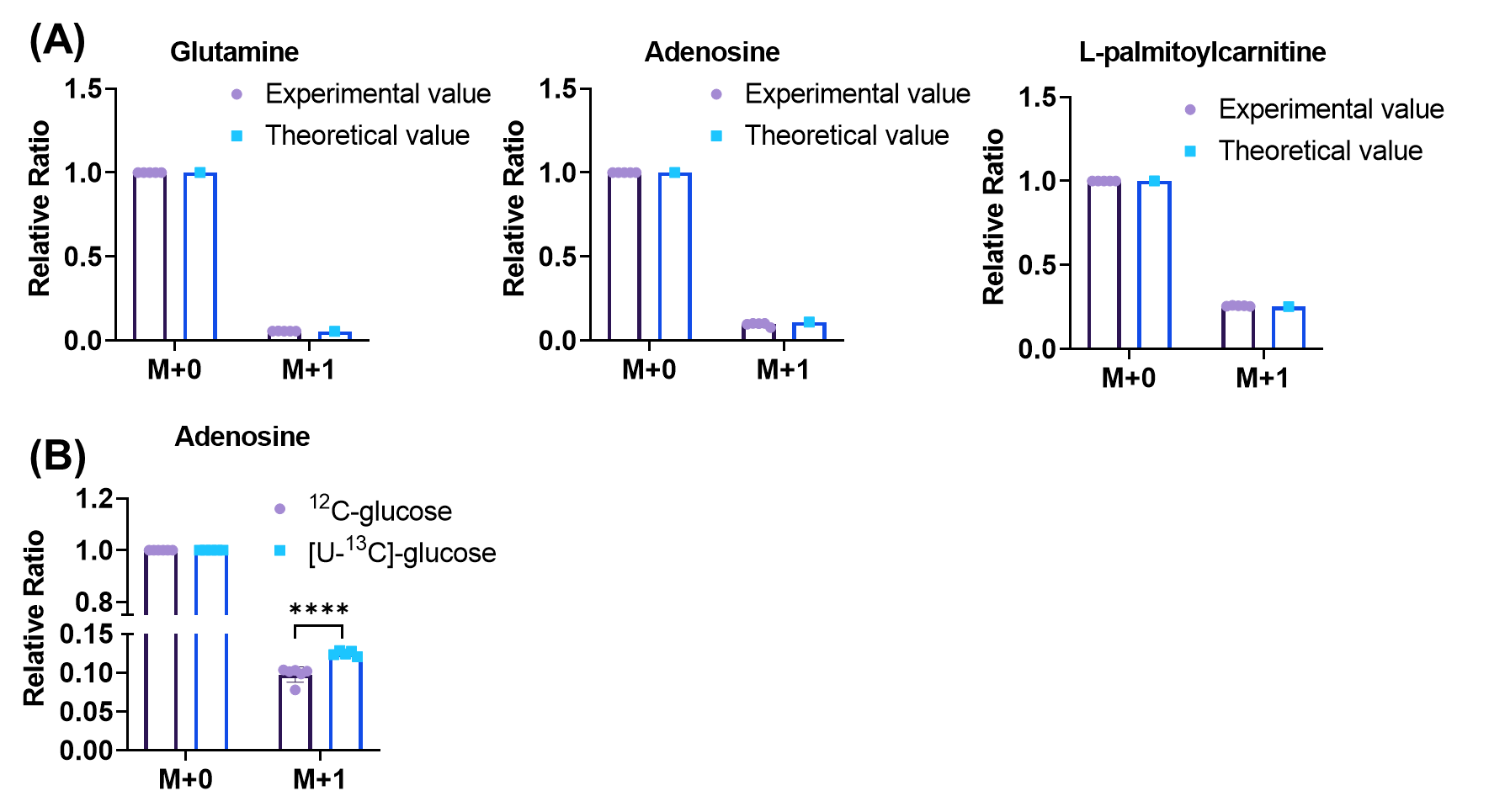


**Supplementary figure 1: The comparison of experimental and theoretical values of M+1 relative abundance of representative metabolites analyzed by orbitrap-based mass spectrometer.** (A) The experimental and theoretical values of M+1 abundance of representative metabolites. (B) The abundance of adenosine M+1 (relative to M+0) in cells treated with or without [U-^13^C]-glucose. The relative ratio of M+0 is set as 1 and M+1 abundance is normalized to M+0 abundance. The data presented in this figure was without natural abundance correction.

******

**Supplementary figure 2: The mass isotopologue distribution of [U-^13^C]-glutamine detected in glutamine free RPMI 1640 medium supplemented with [U-^13^C]-glutamine.** The data presented in this figure was without natural abundance correction.

**
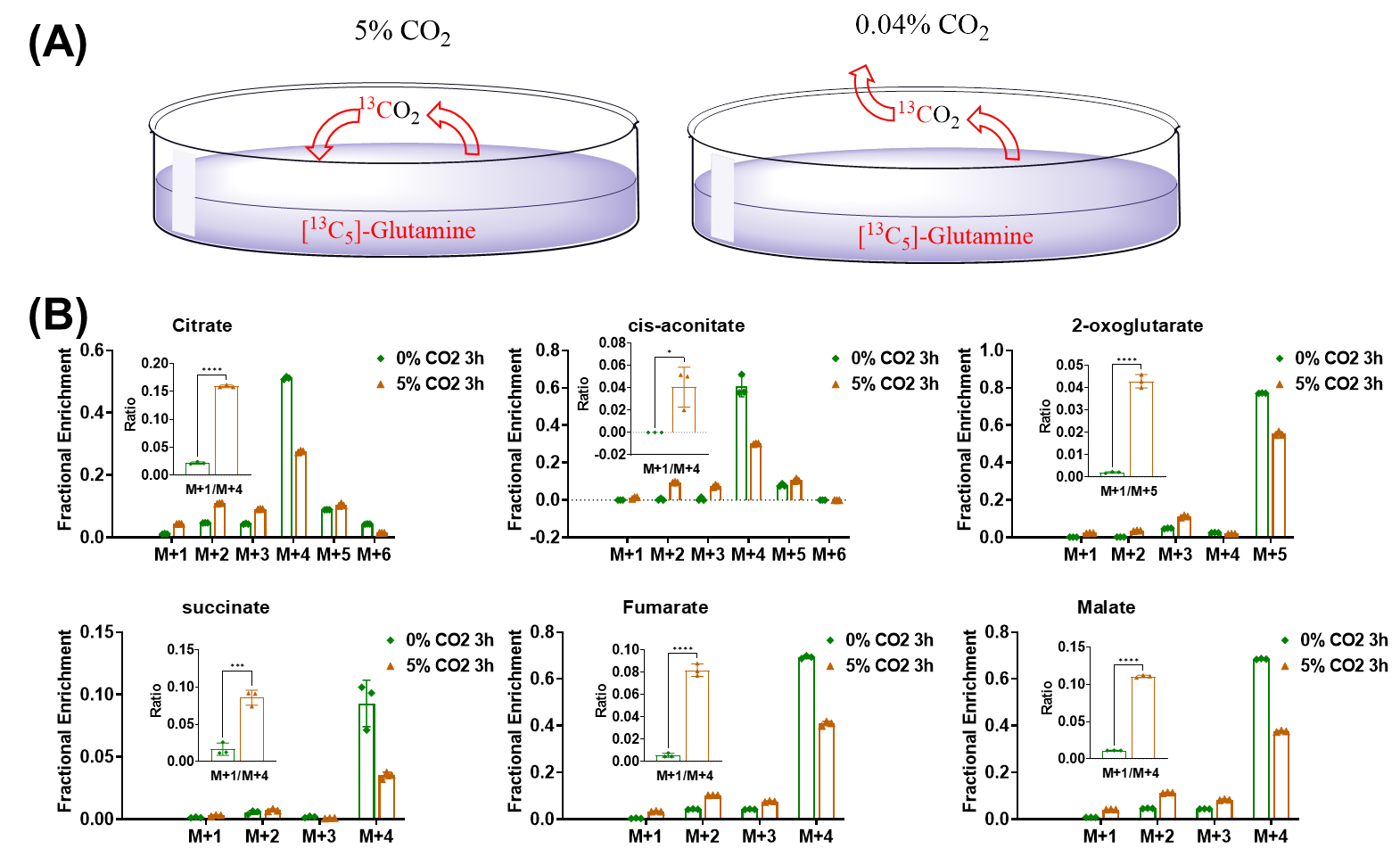
**

**Supplementary figure 3: The effect of exogenous CO_2_ on M+1 labeling in sarcoma cells.** (A) Schematic of in vitro tracing analysis. (B) ^13^C labeling patterns of metabolites from sarcoma cells (in vitro) incubated in [U-^13^C]-glutamine medium in the presence or absence of CO_2_ for 3 hrs. M+1/M+4 ratio (in the insert) was used to account for the overall TCA activity perturbations due to medium changes. Data are presented as mean ± SD of three replicates and after natural abundance correction.

***
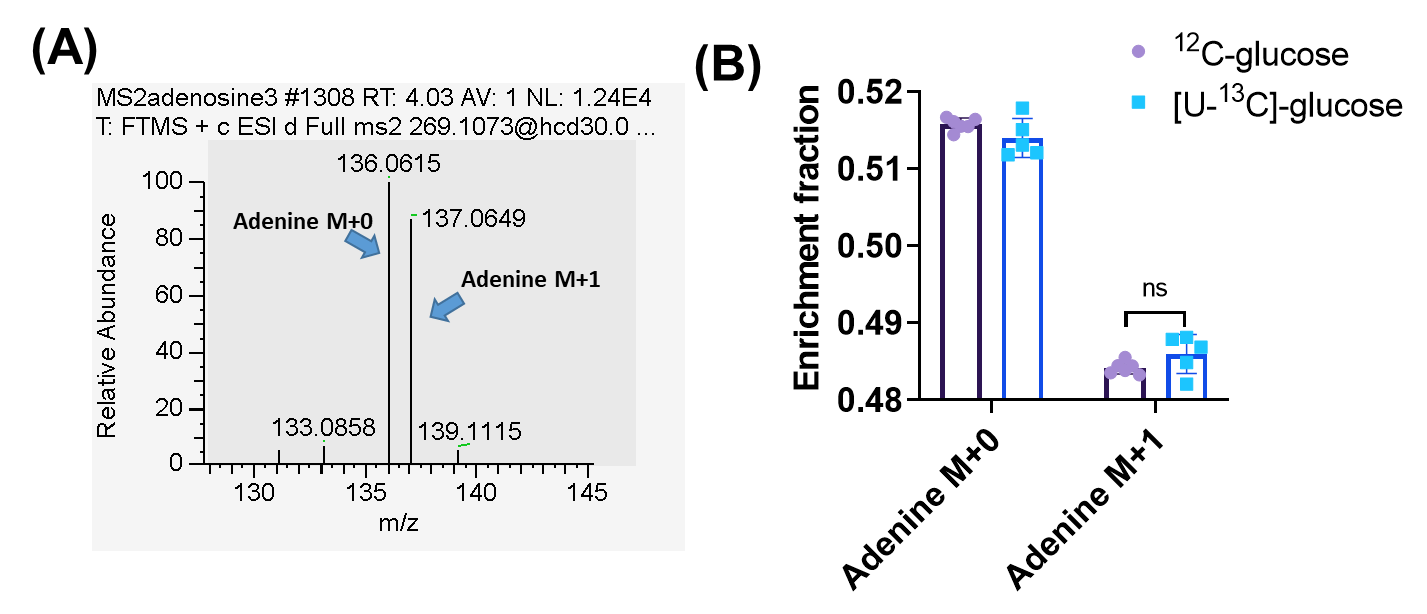
***

**Supplementary figure 4: MS2 analysis of adenosine M+1.** (A) The MS2 spectrum of adenosine fragments. (B) the relative ratio of daughter ion adenine M+0 versus adenine M+1 in the absence of tracer (labeled as “^12^C-glucose”) or [U-^13^C]-glucose. Data in (B) are presented as mean ± SD of three replicates. P value was calculated based on Student’s t test, and “ns” denotes p value larger than 0.05. The data presented in this figure was without natural abundance correction.

******

**Supplementary figure 5: The mass isotopologue distribution of succinyl-CoA in sarcoma of mice receiving [U-^13^C]-glucose tracing.** Data are presented as mean ± SD of n=5 mice. Data with and without natural abundance (NA) correction was presented.


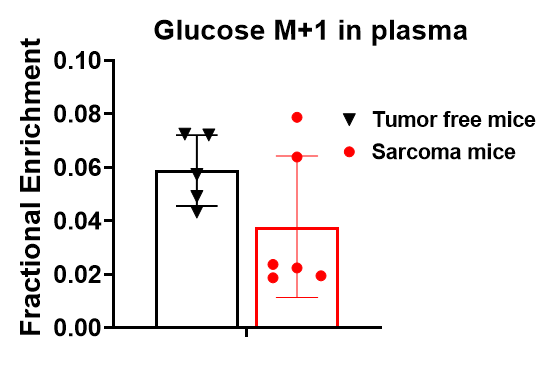


**Supplementary figure 6: Glucose M+1 in the plasma of mice receiving [U-^13^C]-glucose for 3 hrs.** Data presented here was after natural abundance correction.

******

**Supplementary figure 7:** **Pyruvate labeling patterns in sarcoma of mice receiving [U-^13^C]-glucose or [U-^13^C]-glutamine tracing for 3 hrs.** Data are presented as mean ± SD of three replicates and after natural abundance correction.
